# Are Bisphosphonates Effective in the Treatment of Osteoarthritis Pain? A Meta-Analysis and Systematic Review

**DOI:** 10.1371/journal.pone.0072714

**Published:** 2013-09-04

**Authors:** Alison J. Davis, Toby O. Smith, Caroline B. Hing, Nidhi Sofat

**Affiliations:** 1 Department of Rheumatology, Division of Biomedical Sciences, St George’s, University of London, London, United Kingdom; 2 Faculty of Medicine and Health Sciences, University of East Anglia, Norwich, United Kingdom; 3 Department of Orthopaedics, St George’s Hospital, London, United Kingdom; The James Cook University Hospital, United Kingdom

## Abstract

**Objective:**

Osteoarthritis (OA) is the most common form of arthritis worldwide. Pain and reduced function are the main symptoms in this prevalent disease. There are currently no treatments for OA that modify disease progression; therefore analgesic drugs and joint replacement for larger joints are the standard of care. In light of several recent studies reporting the use of bisphosphonates for OA treatment, our work aimed to evaluate published literature to assess the effectiveness of bisphosphonates in OA treatment.

**Methods:**

Literature databases were searched from inception to the 30th June 2012 for clinical trials of bisphosphonates to treat OA pain. Data was appraised and levels of evidence determined qualitatively using best evidence synthesis from the Cochrane Collaboration. The two largest studies were conducted with risedronate in the treatment of knee OA, for which meta-analyses were performed for pain and functional outcomes.

**Results:**

Our searches found 13/297 eligible studies, which included a total of 3832 participants. The trials recruited participants with OA of the hand (n = 1), knee (n = 8), knee and spine (n = 3), or hip (n = 1). Our meta-analysis of the two largest knee studies using risedronate 15 mg showed odds ratios favouring placebo interventions for the Western Ontario and McMaster Universities Arthritis Index (WOMAC) pain (1.73), WOMAC function (2.03), and WOMAC stiffness (1.82). However, 8 trials (61.5%) reported that bisphosphonates improve pain assessed by VAS scores and 2 (38.5%) reported significant improvement in WOMAC pain scores compared to control groups.

**Conclusions:**

There is limited evidence that bisphosphonates are effective in the treatment of OA pain. Limitations of the studies we analysed included the differences in duration of bisphosphonate use, the dose and route of administration and the lack of long-term data on OA joint structure modification post-bisphosphonate therapy. Future more targeted studies are required to appreciate the value of bisphosphonates in treating osteoarthritis pain.

**Trial Registration:**

PROSPERO Register CRD42012002541

## Introduction

Osteoarthritis (OA) is the commonest form of arthritis worldwide and is a major cause of disability and pain. In 1999/2000, OA cost the UK economy nearly £3.2 billion in reduced production [1]. In an ageing population, the prevalence of OA is expected to increase, particularly as there are currently no treatments that delay or halt the progression of disease. The mainstay of treatments currently approved by the UK National Institute for Health and Clinical Excellence (NICE) include pain control with oral or topical analgesic drugs e.g. NSAIDs, opioids, capsaicin, accompanied by physical therapies to maintain function [2]. In people where optimal medical management is insufficient to control symptoms, joint replacement surgery is considered in large weight bearing joints such as the hip and knee. However, pain in people with OA is increasingly documented even after joint replacement surgery. In a systematic review of outcomes, the proportion of people with unfavourable long-term pain following total hip replacement (THR) ranged from 7% to 23%, and 10% to 34% following total knee replacement (TKR) [3]. There is therefore an urgent need to address pain management in people with OA.

In recent years, several studies have investigated the merits of bisphosphonates in the treatment of OA. Traditionally used for the treatment of osteoporosis, bisphosphonates target osteoclast resorption and have been proven to be effective in reducing fracture rates in people with established osteoporosis [4]. Bisphosphonates can attach to hydroxyapatite binding sites on bony surfaces, particularly those which are undergoing active bone resorption [5]. When osteoclasts begin to resorb bone impregnated with bisphosphonate, the bisphosphonate released during resorption impairs the ability of osteoclasts to form the ruffled border, to adhere to the bony surface and to produce the protons required for continued bone resorption [6]. Bisphosphonates also reduce osteoclast activity by reducing osteoclast progenitor development [7]. There is much less information available on how bisphosphonates could be effective in the treatment of OA, although they could act through several mechanisms. Osteoarthritis is known to be associated with altered bone turnover, particularly beneath the thickened subchondral plate, with altered flexibility and increased ability to microfracture [8]. It is therefore possible that bisphosphonates could be used to target the osteoclast-mediated extension of channels from marrow spaces into the non-calcified articular cartilage. It has previously been suggested that the loss of osteochondral integrity in OA lesions can expose the subchondral nerves to pro-inflammatory and pain mediating molecules from the synovial fluid, which could permit growth of sensory nerves into non-calcified articular cartilage [9]. It has also been suggested that osteoclasts may reduce pH at the osteochondral junction, which could lead to sensitization and activation of sensory nerves through ion channels on their peripheral terminals [10]. Recent studies from an animal model of OA also suggested that bisphosphonates could target subchondral bone turnover and synovitis in OA [11].

The most common outcome measures used for pain assessment in musculoskeletal pain studies include the Western Ontario and McMaster Universities Arthritis Index (WOMAC) and visual analogue score (VAS). The scoring systems described have been validated in many studies internationally and now form the core dataset for many studies upon which national bodies base recommendations for care [2]. The WOMAC scale has a widely used standardised questionnaire used by health professionals to evaluate the condition of patients with OA of the knee and hip, including pain, stiffness and physical functioning of the joints [12]. The WOMAC measures five items for pain (score range 0–20), two for stiffness (score range 0–8) and 17 for functional limitation (score range 0–68). Physical functioning questions cover everyday activities such as stair use, standing, bending, walking, sitting and household duties. In contrast, the visual analogue score for pain comprises of a 0–10 rating scale for pain and is well validated internationally in many pain studies [13]. Other studies in our analysis utilised variations of the VAS, also known as the visual rating scale (VRS). Our study has therefore focused primarily on the WOMAC and VAS pain and function outcome measures for meta-analyses and synthesis of studies retrieved since they are the most widely used pain outcome measures in the studies evaluated.

Several international studies have been conducted in recent years to assess whether bisphosphonates can target OA pain. However, to our knowledge, no previous systematic reviews exist on the use of bisphosphonates in OA. We have therefore systematically reviewed the current available literature and discuss our evaluation through a meta-analysis and ‘levels of evidence’ approach where appropriate.

## Materials and Methods

The systematic review methods were conducted in accordance with PRISMA guidelines [14]. The study protocol was published on the PROSPERO registry (Registration Number: CRD42012002541).

### Data Sources, Searches and Extraction

**Figure 1:**
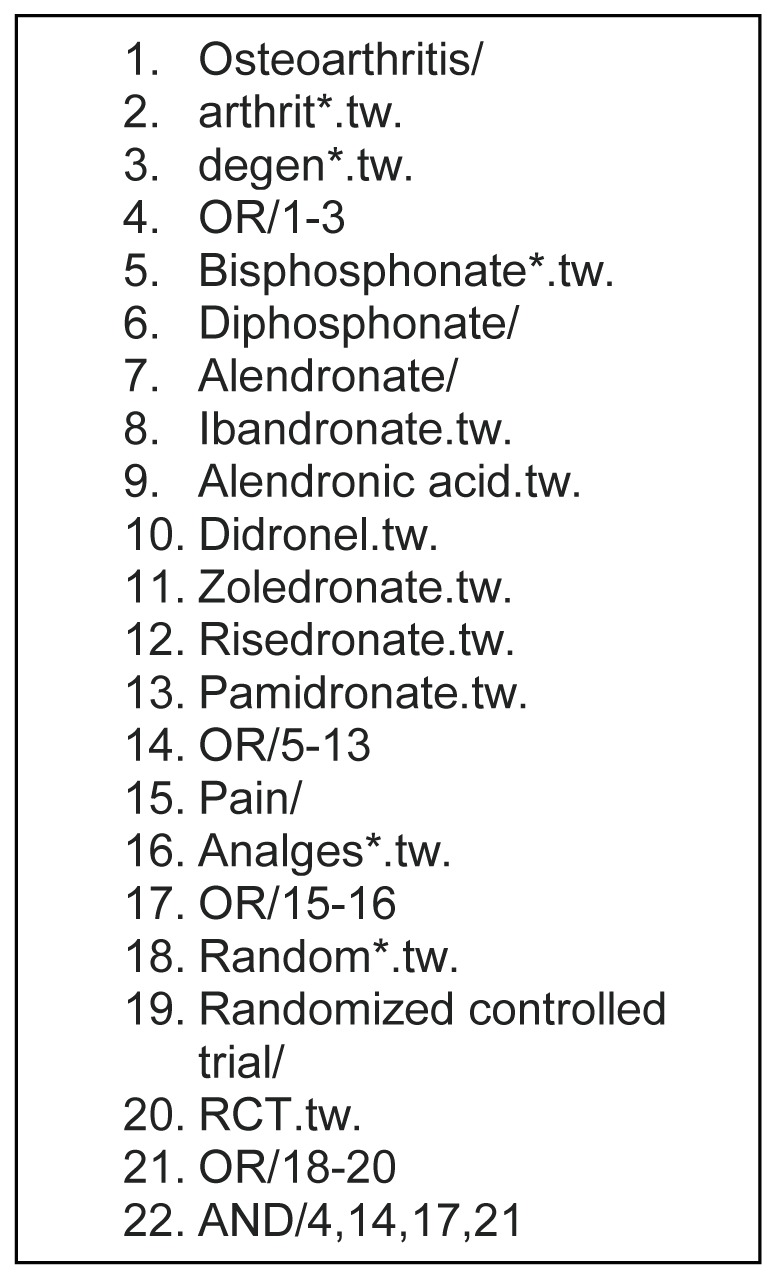
The MeSH terms and Boolean operators used for evaluation.

All searches were conducted from database inception to the 30^th^ June 2012 by one reviewer (AD). The primary search was conducted using the electronic databases AMED, CINAHL, EMBASE, MEDLINE, PUBMED, Web of Knowledge, the Cochrane Library and Biomed Central. Secondary searches were conducted by evaluating the unpublished and grey evidence including: the WHO International Clinical Trials Registry Platform, Current Controlled Trials, DART Europe, rian.ie, British Library Archive & Manuscripts, British Library Theses, OT Seeker, Centre for Reviews and Dissemination, Campbell Library of Systematic Reviews, HMIC, Prospero, ERIC, MIT Theses, WHO Library & Information Networks for Knowledge Database, the United States National Institute of Health Trials Registry, OpenGrey (System for Information on Grey Literature in Europe). The MeSH terms and Boolean operators used are shown in Figure 1.

### Study Eligibility

Two authors (AD/NS) screened the title and abstract of the studies identified from the database searches using the following criteria: randomised controlled trials (RCT) comparing the use of a bisphosphonate therapy to: a non-treatment control, a placebo, another bisphosphonate therapy or to an alternative pharmacological intervention. Trials where participants presented with symptomatic OA of any anatomical region, such as knee, hips, spine and hand were included. Studies where participants had primarily back pain and/or spinal OA were not included. This is justified since the relationship between radiographic OA and back pain is unclear [15]. Any dosage or delivery method and follow-up periods were considered in the study. There were no limits on severity or duration of OA, nor language of study. Exclusion criteria included *in vitro*, animal studies and populations where the predominant pathology was not OA.

### Data Extraction

Data from all eligible papers was extracted by one author (AD) and independently verified by two reviewers (NS, TS). Data extracted included: therapy, diagnosis (including diagnostic criteria for OA), inclusion/exclusion criteria, sample size, cohort age, gender, duration of intervention, primary and secondary outcome measurements, follow-up period, and outcomes recorded at each follow-up period.

### Outcome Measurements

The primary outcome of this evaluation was perceived pain, measured using validated pain assessment scoring systems including visual analogue scale (VAS), a visual rating scale (VRS) or evaluation with the Western Ontario and McMaster Universities Arthritis (WOMAC) index. Secondary outcomes included the assessment of joint structural changes, indirect pain (such as assessment via skin impedance studies), blood and urine analyses of cartilage and bone turnover biomarkers e.g. collagen type 1 cross-linked C-telopeptide (CTX-II) and collagen type 1 cross-linked N-telopeptide (NTX-I) and use of analgesics post-therapy.

### Assessment of Study Quality

Each of the included studies were assessed for methodological quality using the Critical Appraisal Skills Programme (CASP) guidelines [16]. This tool has been adopted widely in previous reviews of musculoskeletal clinical studies [17], [18]. Each included paper was scored by one reviewer (AD) and independently verified by a second (TS). Any disagreements in appraisal score were discussed and resolved by a third reviewer (CH).

### Data Analysis

Study homogeneity was assessed visually by examining the data extraction tables. This identified considerable heterogeneity in relation to the anatomical regions assessed, the type of bisphosphonate and dosage administered, and the comparative intervention used. There was also considerable variability with respect to the follow-up periods assessed. However, for the two studies of bisphosphonates in knee OA, a meta-analysis was appropriate and performed since the numbers were large enough for comparison with between-study homogeneity as previously described [19]. Briefly, statistical heterogeneity was evaluated with Chi^2^ and I^2^ statistical tests. When Chi^2^ equated to p≤0.10 and I^2^≥20%, a random-effects statistical model was undertaken. When Chi^2^ equated with p>0.10 and I^2^ was <20% a fixed–effects model was used. For continuous outcomes, mean difference (MD) or standardised mean difference (SMD) was calculated, with corresponding 95% confidence interval (CI). For each analysis, this was also analysed through a corresponding forest-plot. All statistical analyses were conducted on RevMan version 5.1 (Review Manager (RevMan) [Computer program] Copenhagen: The Nordic Cochrane Centre, Cochrane Collaboration, 2011).

For the remaining studies, a qualitative approach to data synthesis was performed to assess the level of evidence of each included study [19]. The approach described is an alternative to pooling of association sizes when the included studies are heterogeneous and has also been utilised in other systematic reviews in OA [20], [21]. The synthesis was conducted and rated as suggested by the Cochrane Collaboration at five levels: strong; moderate; limited; conflicting; and no evidence. The primary outcome assessed was change in pain measures.

Sensitivity analyses by defining other cut-offs (median score of all studies instead of mean) of high quality studies were performed. A single trial that investigated multiple features (pain and structural change in OA) which were reported in multiple papers, was counted as a single study.

## Results

### Search Strategy

**Figure 2:**
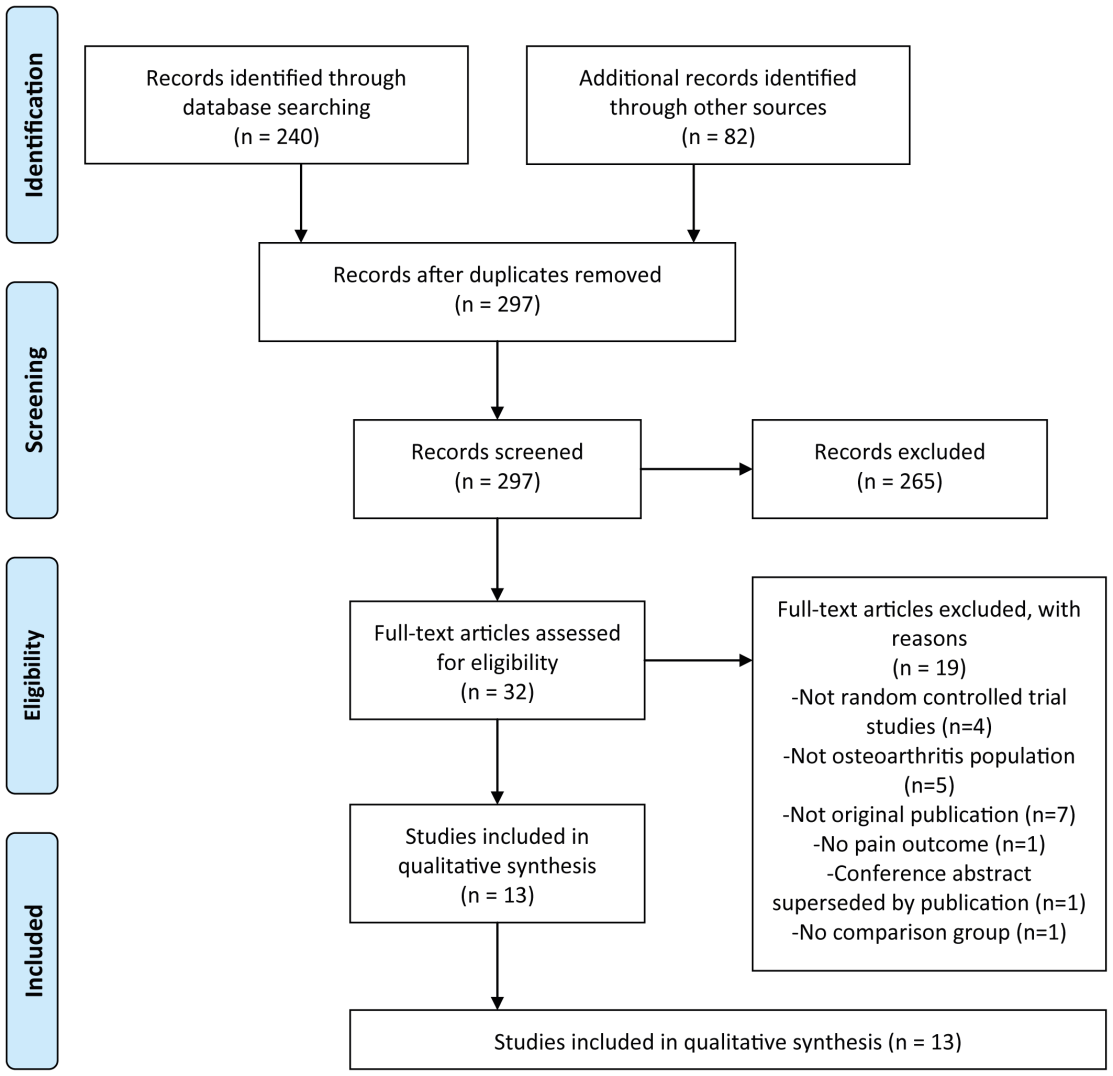
PRISMA flow diagram utilised for systematic review.

The results of the literature search strategy identified a total of 322 studies (Figure 2). A total of 13 studies were deemed eligible and included in this systematic review. This included the removal of duplicates (25 studies), the removal of studies deemed non-eligible based on title and abstract screening (265 studies) and a final 19 studies considered ineligible due to: not being a RCT study (n = 4); studying a non-OA population (n = 5); not being an original publication (n = 7); presenting no pain outcome (n = 1); being a conference abstract that has since been superseded by a full publication (n = 1); or having no comparison group (n = 1).

### Characteristics of Included Studies

Of the 13 eligible studies, one study investigated bisphosphonates in the treatment of hand OA [22], eight studies focused on knee OA and bisphosphonates [23]–[30] whilst three studies recruited people with knee and back/spinal OA [31]–[33]. One study investigated hip OA and bisphosphonates [34].

### Study Quality Assessment

The results of the CASP appraisal illustrate that the current evidence-base presented with a number of significant methodological limitations (Table 1). Strengths of the current evidence-base include a clear definition of the study populations (100%), construction of clear research questions (92%), definition of OA and its diagnosis (77%) and random allocation of participants to intervention received (92%). However, a number of methodological weaknesses limited the quality of the evidence-base. Only five studies (38%) blinded the assessor to group allocation. A total of four studies (31%) did not account for all participants at the end of the study, with only nine studies (69%) assessing for potential baseline imbalance prior to commencing study interventions. A total of 10 studies (77%) appropriately analysed their data and 11 studies (85%) appropriately interpreted their data following analysis.

**Table 1:** Table showing critical appraisal assessment using the CASP tool.

Study	Fujitaet al (2001)	Carbone et al (2004)	Spectoret al(2005)	Bingham et al(2006)	Fujita et al (2008)	Kawasaki et al (2008)	Rossini et al (2009)	Fujita et al (2009)	Jokar et al (2010)	Fujita et al (2011)	Laslett et al (2012)	Nishii et al (2012) Abstract	Saviola et al (2012)
A	✓	✓	✓	✓	✓	✓	✓	×	✓	✓	✓	✓	✓
B	✓	✓	✓	✓	✓	✓	✓	✓	✓	✓	✓	✓	✓
C	×	×	✓	✓	✓	✓	✓	×	✓	×	✓	✓	✓
D	✓	×	✓	✓	✓	✓	✓	✓	✓	✓	✓	✓	✓
E	×	×	✓	✓	✓	✓	✓	✓	✓	✓	✓	N/C	×
F	×	×	✓	✓	×	×	×	×	✓	×	✓	×	×
G	×	✓	✓	✓	✓	✓	✓	×	✓	**×**	✓	✓	✓
H	×	✓	×	×	×	×	✓	×	×	×	✓	×	×
I	✓	✓	✓	×	✓	✓	✓	✓	×	✓	✓	×	✓
J	✓	✓	✓	✓	✓	✓	✓	✓	×	✓	✓	×	✓
K	✓	✓	✓	✓	✓	✓	✓	✓	✓	✓	✓	✓	✓
**Total**	**6**	**7**	**10**	**9**	**9**	**9**	**10**	**6**	**8**	**7**	**11**	**6**	**8**

CASP tool criteria.

A - Was a clearly focused question stated?

B - Was the study population clearly defined?

C - Were all patients who entered the study fully accounted for at its conclusion?

D - Was the assignment of treatment to patients randomised?

E – Was each treatment group similar at the start of the study?

F - Were the patients blinded to their treatment?

G- Was the criteria for OA diagnosis clearly defined?

H - Was the assessor defined?

I - Was there appropriate analysis of results?

J – Was there appropriate interpretation of results?

K - Were the results applicable to clinical practice?

### Study Demographics

The study demographics, clinical study drug use and outcomes are presented in Table 2. In total 3832 participants were reviewed, with a mean age range from 47 to 75 years. In the studies that documented gender, the cohort consisted of 80% women, identified in nine studies. A variety of bisphosphonates were assessed: clodronate in two studies [22], [26] alendronate in four studies [25], [29], [32], [34] risedronate in five studies [23], [24], [27], [30], [32] etidronate in two studies [30], [32] and one study assessed zoledronic acid [28]. Follow-up periods ranged from five weeks to 24 months.

**Table 2:** Characteristics of included studies (listed in chronological order since time of publishing).

Study Authors	Study design	Joint region	Diagnostic criteria	Inclusion criteria	Exclusion criteria	Time (m)	Study drug(s)	Outcome Measure
Fujita et al (2001)^31^	Randomised, non-blinded placebo controlled trial (NBRCT)	Knee/Back	Radiographchange	N = 20 Age: 66 yrs Degenerative joint disease and/or spondylosis deformans with pain	Not stated	12	Doses of etidronate: 0 mg/d i.e. No drug (Gp A), 66 mg/d (Gp B), 133 mg/d (Gp C), 200 mg/d (Gp D)	Visual rating scale, VRS 0–3; Skin impedance
Carbone et al (2004)^25^	Cross sectional cohort study (CS)	Knee	KL (x-ray); WORMS(MRI)	N = 253 69–81 yrs	Multiple bisphosphonate usage Calcitonin users	36	Estrogen (n = 178) Raloxifene (n = 18) Alendronate (n = 57) Drug doses not recorded	Modified WOMAC
Spector et al 2005)^24^	Prospective, randomised, double-blind, placebo-controlled trial (RCT)	Knee	Mild to moderate OAaccording to ACR criteria	N = 231 40–80 yrs OA in ≥1 knee presence of daily pain for ≥1/3 months being ≥50 OR having morning stiffness OR knee crepitus 1 osteophyte Male, Female	Other rheumatic disease, use of hyaluronic acid, injury or diagnostic arthroscopy in previous 6 months, history of knee surgery, intra-articular corticosteroids in previous 6 months, presence of non OA pain, use of bisphosphonate in previous 12months	12	Risedronate 5 mg/d (n = 80) vs. Risedronate 15 mg/d (n = 71) vs. Placebo (n = 80)	WOMAC; PGA (Patient global assessment) Use of analgesics Structural change by JSN Urinary NTX-1, CTX-II
Bingham et al (2006)^23^	Prospective, randomised, double-blind, placebo-controlled trial (RCT)	Knee	Mild-moderate kneeOA with 2–4 mm JSWOA in ≥1 knee dailypain for ≥1/3 mths,being ≥50 ORmorning stiffness ORknee crepitus, 1osteophyteon x-ray	N = 1916; 40–80 yrs Male, Female	Other inflammatory arthritis BMI ≥40 kg/m^2^ Cancer in last 10 yr, tetracycline use in last 6 months, intra-articular injection of corticosteroids or hyaluronan prep in last 3 months, calcitonin or fluoride use in last 6 months, bisphosphonate use in last 12 months, or 60 days ever	24	Risedronate 5 mg/d (n = 493) Risedronate 35 mg/wk (n = 244) Risedronate 50 mg/wk (n = 218) Risedronate 15 mg/d (n = 466) Placebo (n = 475)	WOMAC; PGA Radiograph progression Urinary CTX-II
Fujita et al (2008)^30^	Randomised non-blinded case-control study (NBRCC)	Knee/Back	Radiologic OA with KLscore ≥2	N = 40 Age >50 Patients with OA and osteoporosis	Other rheumatic disorder, endocrine, renal, metabolic disease	6	Risedronate 2.5 mg/d (n = 20) Elcatonin weekly 20 units im (n = 20)	Visual rating scale VRS 0–3; Skin impedance
Kawasaki et al (2008)^27^	Randomised open label (ROL)	Knee	ACR criteria	N = 94 Post- menopausal females	Other inflammatory diseases	18	Risedronate 2.5 mg/d+exercise (n = 31) Glucosamine 1500 mg/d+exercise (n = 33) Exercise alone (n = 30)	WOMAC; Radiograph progression Urinary NTX-I
Rossini et al (2009)^26^	Randomised partially blinded trial (PBRCT)	Knee	ACR criteria for OA	N = 145; Age 50–75 yrs KL grade ≥2 Baseline VAS ≥40 Functional disability ≥3 on scale 1–4	Other joint diseases, anti-coagulants, corticosteroid or chondroprotective therapy, viscosupplementation last 3 months, major surgery to knee, previous arthroscopy last 6 months, allergy to experimental preparations.	1	Clodronate (n = 117) 0.5 mg 1x IA week for 4 weeks, 1 mg; 1x IA weekly for 4 weeks, 2 mg 1x weekly for 4 weeks, 1 mg 2x IA weekly for 2 weeks Hyaluronic acid 20 mg IA 4 weeks (n = 28)	VAS Pain; Lequesne index Joint extension and mobility scores Paracetamol intake
Fujita et al (2009)^32^	Case-control study, non-randomised (NRCC)	Knee/Back	Radiograph OA withKL score ≥2	N = 199; Mean age 57	Not stated	7	E: Etidronate 200 mg/d+calcium 900 mg/d (n = 50) A: Alendronate 5 mg/d+calcium 900 mg/d (n = 49) R: Risedronate 2.5 mg/d+calcium 900 mg/d (n = 50) P: Calcium 900 mg/d (n = 50)	VRS; Skin impedance Urinary NTX-1
Jokar et al (2010)^28^	Randomised, double-blind, placebo-controlled study (RCT)	Knee	Baseline; WOMAC ≥2, ACR criteria	N = 37 Mean age 47	Secondary OA, Arthroscopy or surgery in target knee within last 6 months, intra-articular treatment within last 6 months, other chronic inflammatory disease, previous GI problems, allergies to bisphosphonates, risk factors for osteoporosis	6	Alendronate 70 mg weekly (n = 18) Placebo (n = 19)	WOMAC
Fujita et al (2011)^33^	Randomised case-control study (RCC)	Knee/back	Radiologic OA with KL score ≥2	N = 38 Age >50 yrs	Other systemic endocrine, metabolic, renal &/or rheumatic disease which generates pain	6	Elcatonin 20 units, im injection weekly (n = 18) Risedronate 2.5 mg/d (n = 20)	VRS; Skin impedance
Laslett et al (2012)^28^	Randomised double-blind placebo-controlled trial (RCT)	Knee	ACR criteria knee OA VAS ≥40 mm ≥1 BML on MRI	N = 59; Age 50–80 yrs	Abnormal blood tests, prior diagnosis of cancer <2yr with ongoing treatment, previous bisphosphosphonates, history or non-traumatic iritis or uveitis, severe knee OA	12	Zoledronic acid 5 mg/100 ml (n = 31) Placebo (n = 28)	VAS; Knee injury and OA outcome score KOOS; BML size analysis
Nishii et al (2012)^ 34^	Randomised case-control study (RCC)	Hip	RadiographOA with K/L ≥2	N = 51 Age 30–90 yrs	No previous bisphosphonate use	24	Cacium lactate 600 mg/d (n = 18) Alendronate 35 mg/week (n = 33)	VAS; Radiograph progressionUrinary NTX-I, CTX-II, MRI
Saviola et al (2012)^22^	Non-randomised case control study (NRCC)	Hand erosive OA	VAS >4/10	45–75 years Male, Female	Inactive hand erosive OA (EOA), EOA with irreversible damage, presence of renal, cardiovascular, neurologic, psychiatric, neoplastic, retinal diseases, other rheumatic disease	24	Clodronate 300 mg iv 7 days followed by 100 mg im for 14 days every 3 months (n = 24) vs HCQ 400 mg daily for 30 days followed by 200 mg daily for 11 months (n = 14)	VAS; Hand strength Number of swollen and painful joints

### Risedronate and Knee OA: Meta-analysis

**Figure 3:**
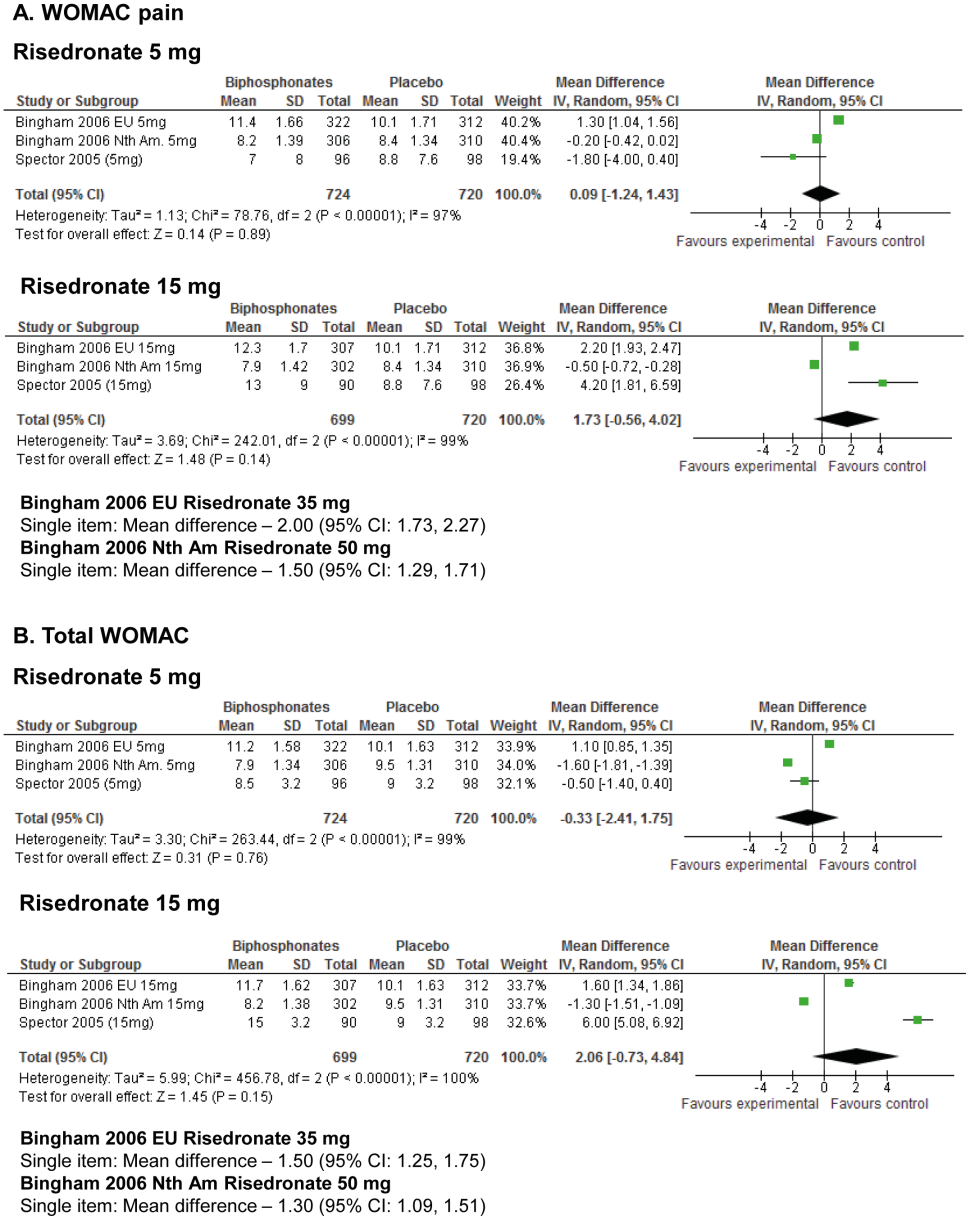
Meta-analysis of knee OA studies for WOMAC pain and total WOMAC outcomes.

**Figure 4:**
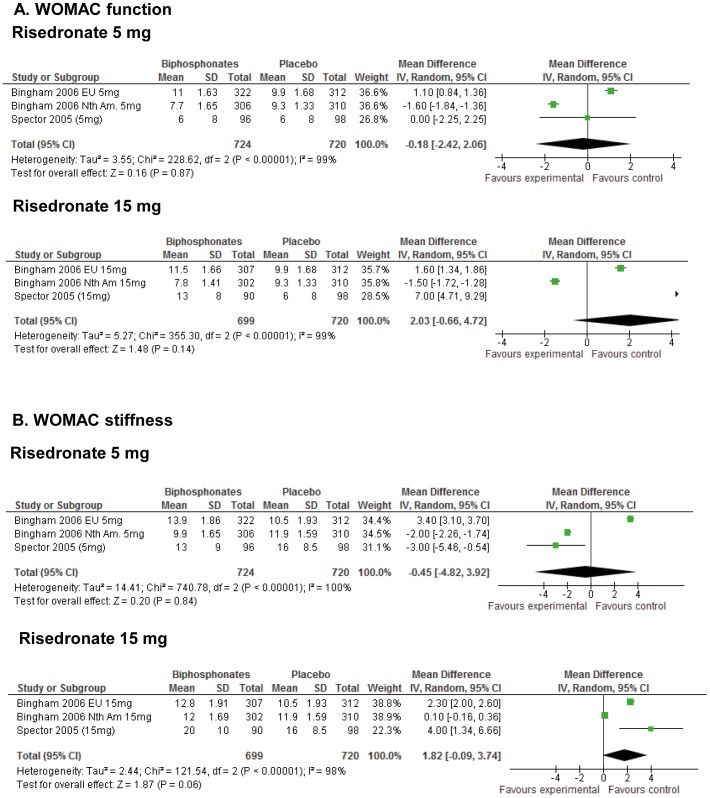
Meta-analysis of knee OA studies for WOMAC function and stiffness scales.

Two large randomised controlled studies have been conducted of risedronate use in knee OA [23], [24]. The studies reported by Bingham *et al.* [23] and Spector *et al.* [24] formed the basis of a meta-analysis. The results of the meta-analyses are presented in Figure 3 & 4. This identified that for WOMAC pain, there was no statistically significant difference between risedronate and placebo for 5 mg daily (OR: 0.09; 95% CI: −1.24, 1.43; Figure 3A); and 15 mg weekly (OR: 1.73; 95% CI: −.56, 4.02; Figure 3A). No significant effects were observed for higher doses of risedronate 35 mg and 50 mg weekly respectively on single-item analyses.

Further secondary analyses were conducted using the total WOMAC score. This reported no statistically significant difference between risedronate and placebo interventions at 5 mg daily (Odd Ratio (OR): −0.33; 95% Confidence Intervals (CI): −2.41, 1.75; Figure 3B), or 15 mg weekly (OR: 2.06; 95% CI: −0.73, 4.84; Figure 3B). At higher risedronate doses of 35 mg and 50 mg weekly where only a single-item analysis could be performed, no significant improvement in WOMAC outcome was observed.

Similarly, there was no statistically significant difference for risedronate on WOMAC function at a 5 mg daily dose (OR: −0.18; 95% CI: −2.42, 2.06; Figure 4A) or 15 mg weekly dose (OR: 2.03; 95% CI: −0.66, 4.72; Figure 4A). At higher doses of 35 mg and 50 mg weekly risedronate, no significant effects were observed versus placebo. WOMAC stiffness subscale also demonstrated no statistically significant differences between the groups at 5 mg daily dose (OR: −0.45; 95% CI: −4.82, 3.92; Figure 4B), and 15 mg weekly dose (OR: 1.82; 95% CI: −0.09, 3.74; Figure 4B). Our meta-analysis results show that for standardised WOMAC outcomes, no significant effects were observed at any dose of risedronate compared with placebo.

In addition, there were no statistically significant differences or trends were noted for any dose of risedronate. Similarly there was no difference between the five groups with respect to radiographic joint space narrowing, joint space width, or osteophyte formation during the 24 month follow-up (p>0.05). There was a significant decrease in collagen type 1 cross-linked N-telopeptide levels, or NTX-1, which was dose-dependent in the risedronate group during follow-up (p<0.05).

Spector *et al*. [24] reported no statistically significant difference in loss of joint space width (p = 0.28) between the different intervention groups and placebo during a 12 month follow-up period. There was a statistically greater improvement in patient global assessment (p<0.001) and reduced use of walking aids (p = 0.009) between the 15 mg weekly risedronate group compared to the placebo group at 12 months. This was not evident in the 5 mg/day cohort. In additional biomarker analysis, there was a statistically significant decrease in mean urinary c-terminal crosslinking telopeptide of type II collagen (CTX-II; marker of cartilage turnover) and N-terminal crosslinking telopeptide of type I collagen (NTX-I; marker of bone absorption) values in the 15 mg risedronate weekly group compared to the placebo group (p<0.05).

### Bisphosphonates and Knee OA: Narrative Research Synthesis

#### Estrogen vs. Raloxifene vs. Alendronate vs. No treatment

Carbone *et al*. [25] compared the use of alendronate to oestrogen and raloxifene in a study of 818 women. The authors found no significant association between overall use of anti-resorptive drugs and the presence of knee pain and radiographic changes of OA of the knee (p>0.05). Use of alendronate, but not oestrogen, was associated with less severity of knee pain as assessed by WOMAC scores. Whilst there was no statistically significant difference between the intervention groups in WOMAC score (p>0.05), there were statistically improved scores in the alendronate group compared to the no-treatment group (p<0.05). There was no statistically significant difference between the three groups receiving alendronate, oestrogen or raloxifene in response to the presence or absence of knee symptoms or radiological OA in the tibiofemoral (p≥0.90), patellofemoral (p≥0.12) compartments or the whole knee (p≥0.43). There was no statistically significant difference between the three intervention groups in bone attrition, osteophytes, BML or cartilage lesions (p>0.05).

#### Clodronate (0.5 mg I.A./week; 1.0 mg I.A./week; 2.0 mg I.A./week; 2 I.A injections, 1.0 mg/week) vs. Hyaluronic acid (20 mg/week)

Rossini *et al*. [26] compared the prescription of different dosages of clodronate to hyaluronic acid with people with knee OA. They reported no statistically significant difference between the groups with respect to VAS pain (p>0.05) or mobility scores (p≥0.06) during the initial five weeks. However after adjusting for multiple comparisons and paracetamol use, the authors reported that there was significantly less pain in those allocated to the intra-articular injections 1 mg compared to the hyaluronic acid group (p<0.01). There was no statistically significant difference between the groups with respect to side effects (p>0.05).

#### Risedronate (2.5 mg/day) and Exercise vs. Glucosamine and Exercise vs. Exercise alone

Kawasaki *et al*. [27] reported no statistically significant difference in additive events between their three intervention arms, although an improvement in individual pain scores was seen after 18 months. There was no statistically significant difference between the groups in functional outcomes at 18 months as assessed with the Japan Orthopaedic Association score (p>0.05), VAS pain (p>0.05), WOMAC score (p = >0.05), joint space width (p>0.05) or use of NSAIDS (p>0.05).

#### Zoledronic Acid (5 mg/100 ml) vs. Placebo

Laslett *et al*. [28] compared clinical outcomes between a single in-fusion of zoledronic acid (5 mg/100 ml) to a placebo control group. This trial showed significant improvements in pain using the VAS at six months, which was the primary endpoint of this study. There was a −14.5 mm reduction in VAS at six months. Of note, there was no significant improvement in VAS at three or 12 months. Change in the Knee Injury and Osteoarthritis Outcome Score (KOOS) was not significant at any time point in this study. The authors also reported a reduction in total BML area of greater magnitude in the zoledronate group compared with placebo after six months (−175.7 mm^2;^ 95% CI: −327.2 to −24.3) with a non-statistically significant trend after 12 months (−146.5 mm^2;^ 95% CI: −307.5 to 14.5). With respect to adverse outcomes, the prevalence of adverse events in the zoledronate group was 90%. Of these adverse events, the most common was cold or flu symptoms, which was 78% of the 90% total.

#### Alendronate (oral: 70 mg/week) vs. Placebo

Alendronate (oral: 70 mg/week) was compared to a placebo control in a trial by Jokar *et al.* [29]. The authors reported no statistically significant difference between the two groups at six month follow-up for WOMAC functional score (p>0.05), use of NSAIDs (p = >0.05) frequency of adverse events and side effects (p = >0.05). The most common side-effects were dyspepsia (alendronate = 15%; placebo = 10%) and heart burn (alendronate = 10%; placebo = 10%).

### Bisphosphonates in Hand OA

#### Clodronate (300 mg i.v.) vs. hydroxychloroquine (i.m. 100 mg) at 3 months

One study compared the use of a bisphosphonate with hydroxychloroquine [22]. A statistically significant decrease in pain during the 24 month follow-up in the clodronate group (p = 0.02) was reported, and greater physician and patient global assessment scores in the clodronate group compared to the hydroxychloroquine group (p<0.001).

### Bisphosphonates in Knee and Spine OA

#### Etidronate (66 mg/day; 133 mg/day; 200 mg/day) vs. No-Treatment Control

Fujita *et al*. [30] reported statistically significantly lower VRS subjective pain, and mean fall in skin impedance in the three etidronate dosage groups, compared to the no-treatment control (p<0.001) during the 12 months follow-up period. The authors also reported a positive relationship between the dose of etidronate to bone mineral density and pain, where an increased effect of etidronate was associated with increased bone mineral density and reduced pain (p<0.05).

#### Risedronate (2.5 mg/day) vs. Etidronate (200 mg/day) vs. Alendronate (5 mg/day) vs. No-Treatment Control

A comparison of risedronate, etidronate and alendronate was made to a no-treatment control group in Fujita *et al*. [32] with participants who presented with symptomatic knee and spinal OA. There was a significant reduction in pain as assessed by a fall in skin impedance and by subjective pain response with bisphosphonates compared to the no-treatment control (p<0.001). There was no statistically significant difference between the three bisphosphonate groups (p>0.05). Previously, Fujita *et al*. [31] compared risedronate versus calcitonin in OA in the knee and spine and found similar analgesic outcome measures in both arms of the study (p>0.05).

### Bisphosphonates in Hip OA

#### Alendronate (35 mg/week) vs. Calcium Lactate (600 mg/day)

One study assessed the use of bisphosphonates with hip OA [34]. The use of alendronate (35 mg/week) provided statistically significant better pain reduction compared to those randomised to receive calcium lactate (600 mg/day) at 24 months. There was no statistically significant difference in radiological OA progression (p>0.05).

### Level of Evidence Assessment

Based on the studies evaluated above, we have synthesised levels of evidence by assessing the quality of the studies. Our meta-analysis of the two largest knee OA studies showed no statistically significant difference in pain or functional outcomes assessed by WOMAC with risedronate over placebo arms at doses of risedronate at doses of 5 mg daily, or 15 mg, 35 mg and 50 mg weekly. The remaining studies, which could not be evaluated by meta-analysis, showed that bisphosphonates reduce pain greater than placebo or non-treatment controls in OA in Asian, European and North American populations when assessed by VAS and WOMAC outcomes. There was heterogeneity across the studies analysed, with variability in anatomical position of disease, gender studied, doses, route and frequency of drug administration.

## Discussion

The outcome of our analysis concludes that bisphosphonates demonstrate limited evidence for pain modulation in OA. There is little clinical information available from published studies of the effects of bisphosphonates on the most significant correlates of pain in OA, which include synovitis and bone marrow lesions [35]. The studies assessed highlight that OA is a heterogeneous disease varying with time and may explain why none of the studies reported difference in both synovitis and BML at follow-up over several years. Nonetheless specific studies such as Laslett *et al.* [28] and Nishii *et al*. [34] attempted to examine modulation in BML by bisphosphonates temporally over the course of their clinical studies. Adequate targeting of OA participants to specific time-points in their disease process when such changes may occur would be a valuable addition to the evidence-base or correlation of bisphosphonate use with not only clinical pain outcomes but also progression of BML and/or synovitis. The correlation of pain outcomes including VAS and WOMAC with radiographic correlates of pain such as synovitis and BML are important issues to be considered for future clinical trial designs of bisphosphonates. However, the studies described may only explain a small proportion of pain variance and other mediators, as yet uncharacterised, may be more important mediators of OA pain.

From a safety perspective, regarding the use of bisphosphonates in OA populations, the most common adverse events to bisphosphonate use were gastrointestinal, principally dyspepsia complications. There was no statistically significant difference in these events in those prescribed bisphosphonates compared to non-treatment or placebo. Prescribed medications were otherwise well tolerated with no major significant effect on blood counts, renal and liver function.

Much is yet to be learned with respect to the progression of OA and the relation between level of pain experienced by OA populations in comparison to regions of structural change e.g. joint space narrowing, bone marrow lesion size and synovitis. There is therefore an urgent need for further studies of bisphosphonates in OA in clearly defined subsets, coupled with robust radiographic analysis by cartilage integrity, BML size/composition, synovitis, joint space narrowing and evaluation of clinical biomarkers to more fully evaluate agents that could halt the onset and/or progression of OA.

Future studies targeted at assessing bisphosphonate use for the management of OA would benefit by selecting specific phenotypes of participants in order to assess which groups may benefit most from intervention. Considerations would need to include staging and severity of osteoarthritis, not only perhaps assessed by joint space narrowing, but also to include site and size of bone marrow lesions, degree of synovitis and joint space narrowing, which could be assessed using standardized scoring systems such as the Magnetic Resonance Imaging Knee Osteoarthritis Score (MOAKS) [36]. Since the most widely accepted pain and functional outcome data are recorded using WOMAC and VAS, future trials would need to include such measures in order to make valid comparisons with existing studies. The temporal sequence of the effect of the bisphosphonate intervention also needs to be considered carefully in future studies, with not only pain and functional outcome measures, but also structural modification effects including bone marrow lesion size, synovitis and joint space narrowing measures. Since OA is a chronic long-term disease in which any pharmacological intervention would need to be efficacious and tolerated over a significant period of time, long-term follow-up data beyond one year would also be most helpful in determining long-term effects and tolerability of bisphosphonates in OA.

## Supporting Information

Table S1 PRISMA checklist summary for meta-analysis and systematic review
(DOC)
